# Supplementary material for: Surface-Tethering Enhances Precision in Measuring Diffusion Within 3D Protein Condensates
Source: bioRxiv. 2025 Jun 17:2025.06.11.659185. Preprint. [Version 1] doi: 10.1101/2025.06.11.659185 (PMC12262706; doi:10.1101/2025.06.11.659185)
Supplement: Supplement 1 [file media-1.pdf]

Appendix A. Supplementary material for:

Surface-Tethering Enhances Precision in Measuring Diffusion Within 3D Protein Condensates

Emily R. Sumrall[1,2#], Guoming Gao[1,2,3#], Shelby Stakenas[4], and Nils G. Walter[2,4]

[1]Biophysics Graduate Program, University of Michigan, Ann Arbor, MI 48109, USA

[2]Center for RNA Biomedicine, University of Michigan, Ann Arbor, MI 48109, USA

[3]Division of Biology and Biological Engineering, California Institute of Technology, Pasadena, CA 91125, USA

[4]Department of Chemistry, University of Michigan, Ann Arbor, MI 48109, USA

[#]These authors contributed equally to this work

Correspondence to Nils G Walter: Department of Chemistry, University of Michigan, Ann Arbor, MI 48109, USA. *nwalter@umich.edu (N.G. Walter)*

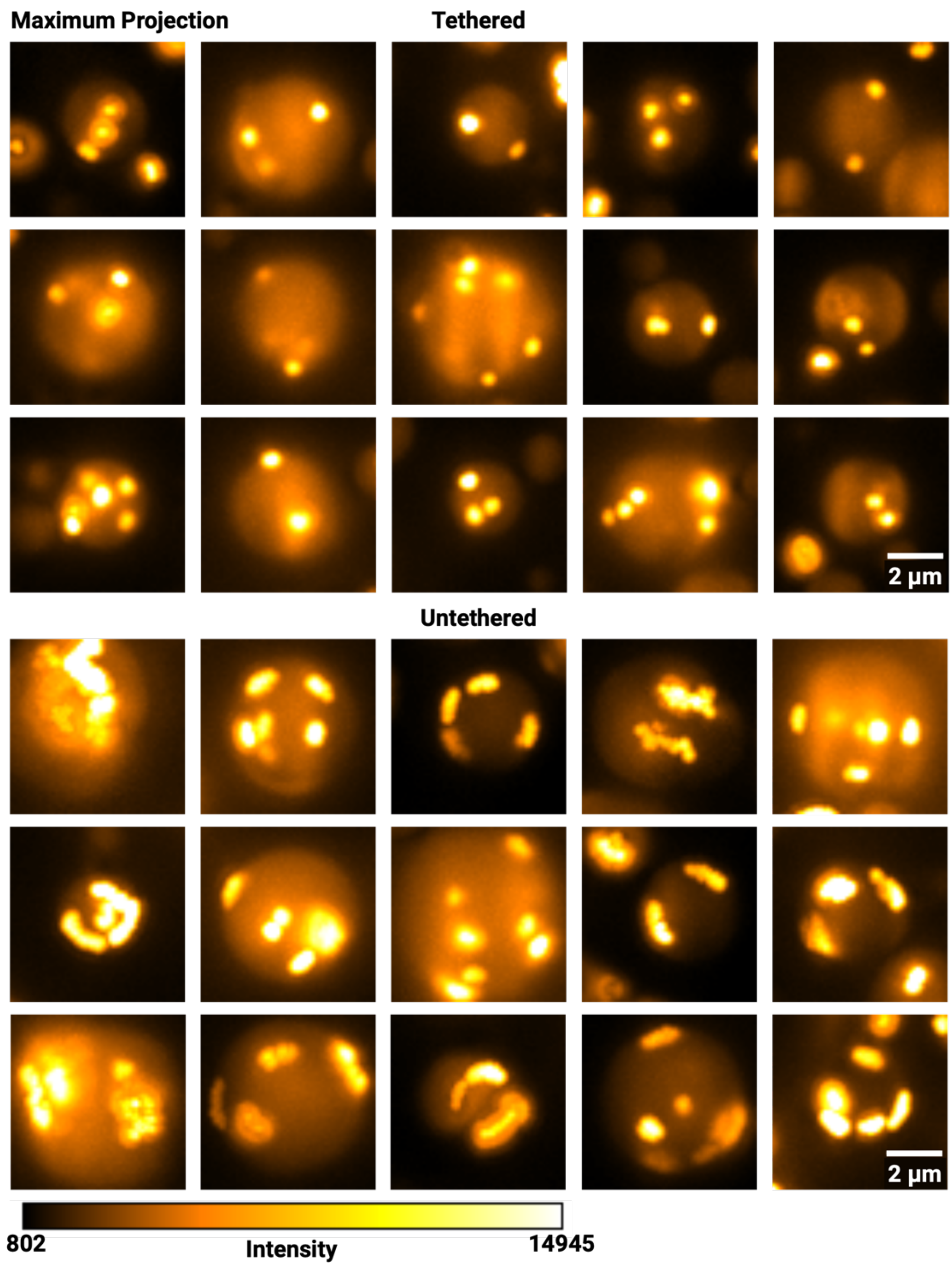

Figure S1 | Maximum projections of fluorescent beads reveal underlying rotation and translation during imaging. 200 nm carboxylated fluorescent beads are immobile at the 10s imaging time using 100 ms frame rate, thus maximum projections of intensity values are a simple and effective approximation for observing rotation or translation of whole condensates. **Tethered |** Maximum projections reveal stationary beads for the duration of imaging and thus indicate stably tethered condensates. **Untethered |** Maximum projections reveal diffusive beads and thus indicate unstable condensates with rotation or translation throughout the duration of imaging.

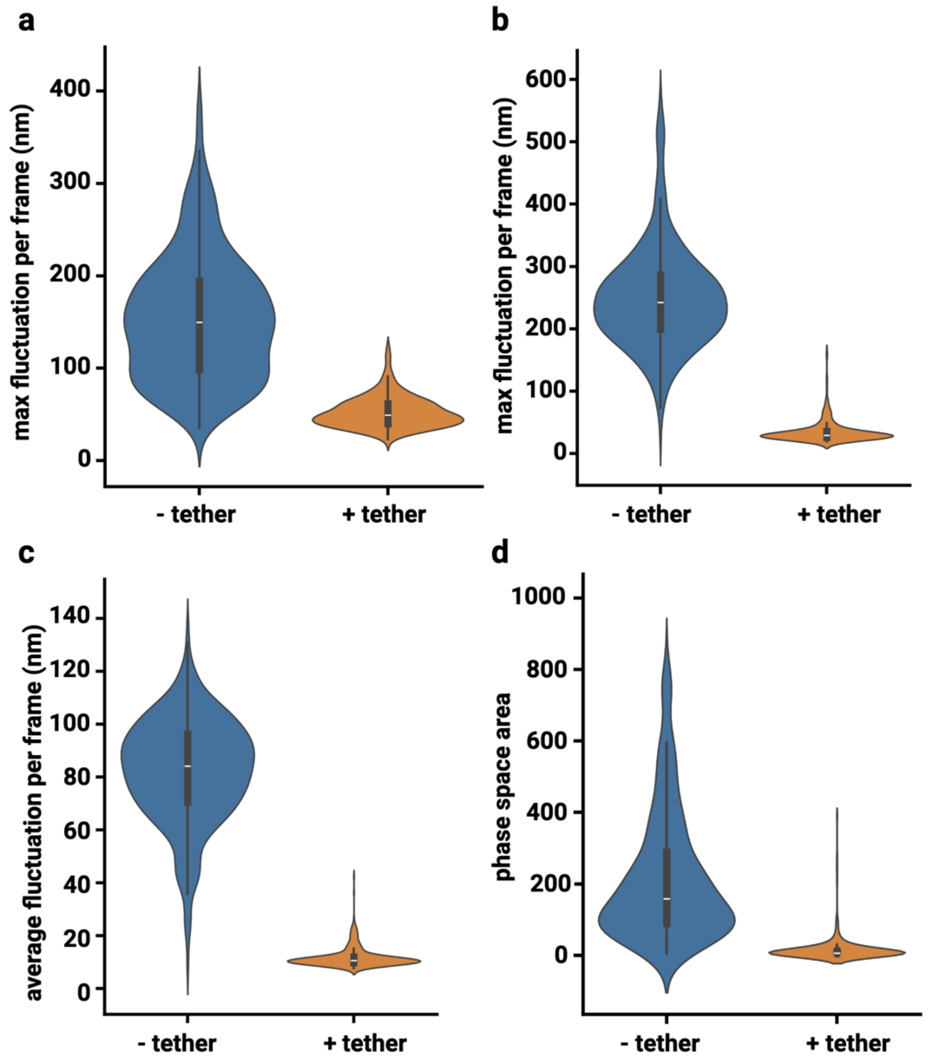

Figure S2 | Fluctuation and rotation characteristics are independent of imaging frequency. 200 nm carboxylated fluorescent beads imaged at 100 ms for 100 frames. **a |** Maximum frame-to-frame localization fluctuations for all condensates in each condition. **b-d |** 200 nm carboxylated fluorescent beads imaged at 20 ms for 500 frames. **b |** Maximum frame-to-frame localization fluctuations for all condensates in each condition. **c|** Average frame-to-frame localization fluctuations for all condensates in each condition. **d |** Phase space area scatter plot of velocity-acceleration correlation reveals distinct physical characteristics for untethered versus tethered condensates. As expected, these results align with Figure 3 which compare the same conditions using a 100 ms frame rate. $N_{untethered}$ = 389, $N_{tethered}$ = 310. Statistics annotation: Mann-Whitney U, ns: $0.05 < p <= 1$, *: $0.01 < p <= 0.05$, **: $0.001 < p <= 0.01$, ***: $0.0001 < p <= 0.001$, ****: $p <= 0.0001$.

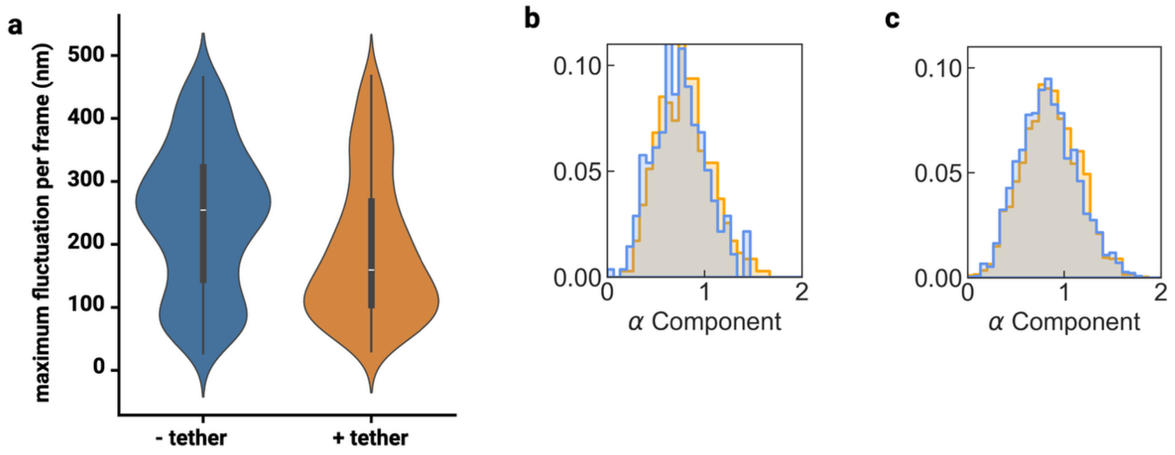

Figure S3 | Extended SPT analysis of FL RNA in untethered or tethered condensates imaged or analyzed with 200 ms frequency. a | Maximum frame-to-frame localization fluctuations for all condensates in each condition. **b |** Distribution of anomalous diffusion component, α. Theoretically, an α value of 1 (or distribution centered around 1) reflects Brownian motion, though this value is often underestimated so a distribution around 0.7 – 1.0 is expected. **Extended SPT analysis of Poly(U)$_{1000}$ RNA in untethered or tethered condensates imaged or analyzed with 200 ms frequency. c |** Distribution of anomalous diffusion component, α.

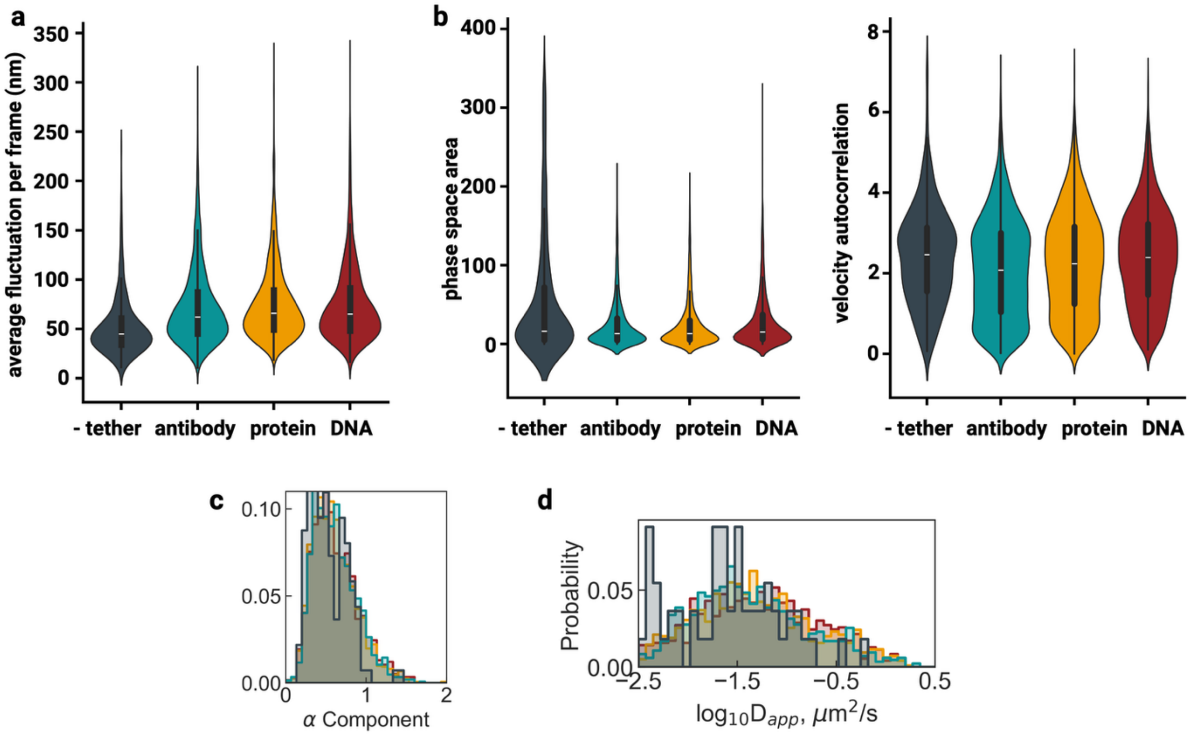

Figure S4 | Extended SPT analysis of FL RNA in antibody-, protein-, or DNA- tethered condensates imaged or analyzed with 20 ms frequency. a | Violin plot of average fluctuation distributions from Fig. 6a, now including the -tether condition. **b |** Violin plots of phase space area and velocity autocorrelation distributions from Fig. 6b, now including the -tether condition. **c |** Distribution of anomalous diffusion component, α. **d |** Histogram distributions of log-transformed apparent diffusion coefficients ($\log_{10}D_{app}$, $\mu m^2/s$) from Fig. 6e, now including -tether condition.

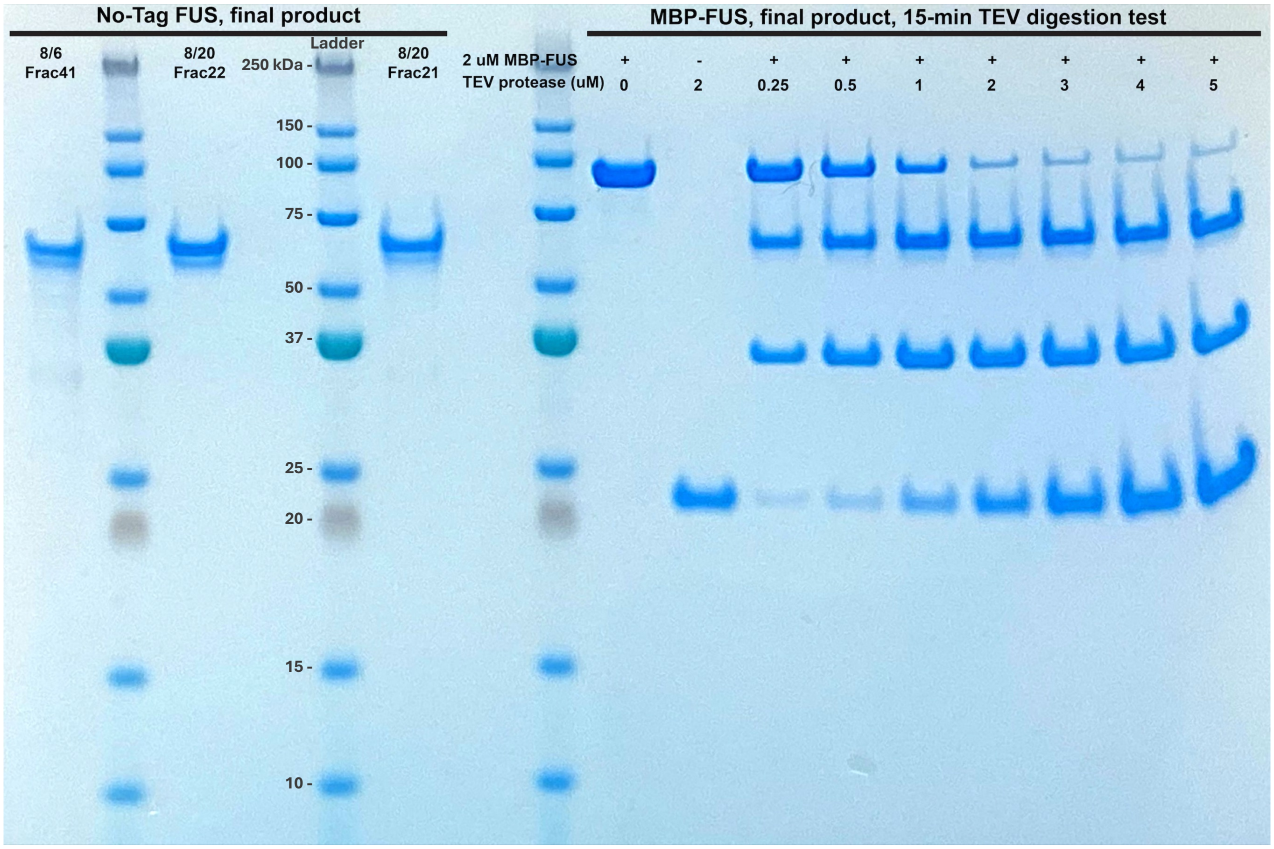

Figure S5 | FUS purification gel. No-Tag FUS | Occupied lanes from left to right: (1) final product fraction of no-tag FUS purification, dominant band running near ~70 kDa as expected, (2) Precision Plus Protein Ladder (Bio-rad), (3) final product fraction of no-tag FUS purification, (4) Precision Plus Protein Ladder (Bio-rad), (5) final product fraction of no-tag FUS purification. **MBP-FUS |** Occupied lanes from left to right: (1) Precision Plus Protein Ladder (Bio-rad), (2) final product fraction of MBP-FUS purification, 0 μM TEV-protease, dominant band running near ~98 kDa as expected, (3) 0 μM MBP-FUS, 2 μM TEV-protease, dominant band running near ~23 kDa as expected, (4-10) 2 μM MBP-FUS with increasing concentrations of 0.25, 0.5, 1, 2, 3, 4, 5 μM TEV-protease respectively, dominant bands running near ~98 kDa for uncleaved MBP-FUS, ~70 kDa for cleaved full length FUS, ~35 kDa for cleaved MBP fragment, and ~23 kDa as expected for TEV protease. For all lanes, signal is from Coomassie Blue staining.

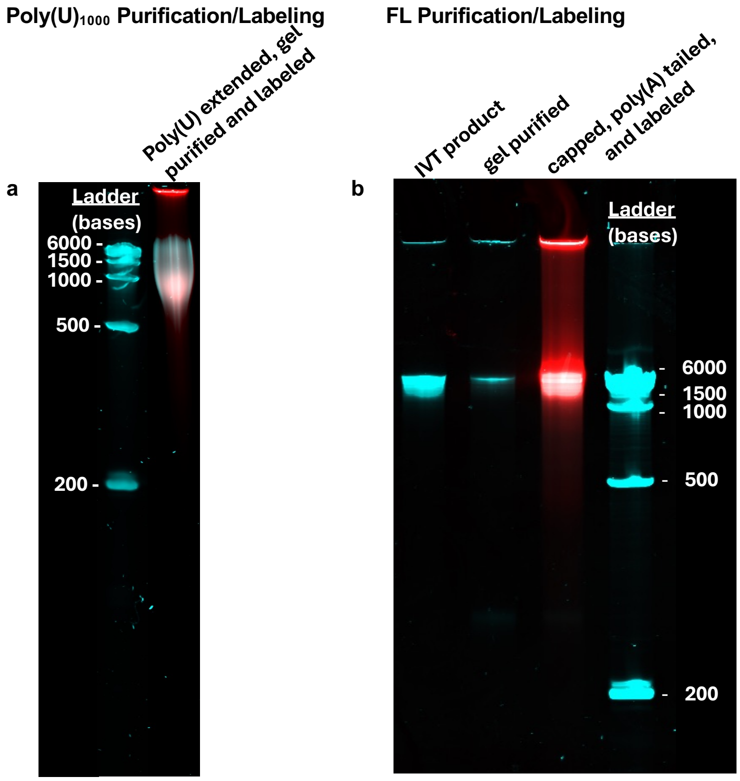

Figure S6 | Poly(U)$_{1000}$ and FL RNA purification Gel. a | Occupied lanes from left to right: (1) RiboRuler High Range (ThermoFisher) ladder for RNA PAGE. (2) Poly(U)$_{1000}$ gel purification product after Poly(U) extension, Azido-ATP addition, and AlexFluor-647 Click-chemistry labeling. **b |** Occupied lanes from left to right: (1) RNA IVT product (2) RNA gel purification product after IVT (3) RNA gel purification product after Azido-ATP addition, Poly(A) tail extension, 5'-OMe cap, and AlexFluor-647 Click-chemistry labeling. (4) RiboRuler High Range (ThermoFisher) ladder for RNA PAGE. For all lanes, cyan represents syber-gold stain signal and red corresponds to AlexaFluor-647 signal.

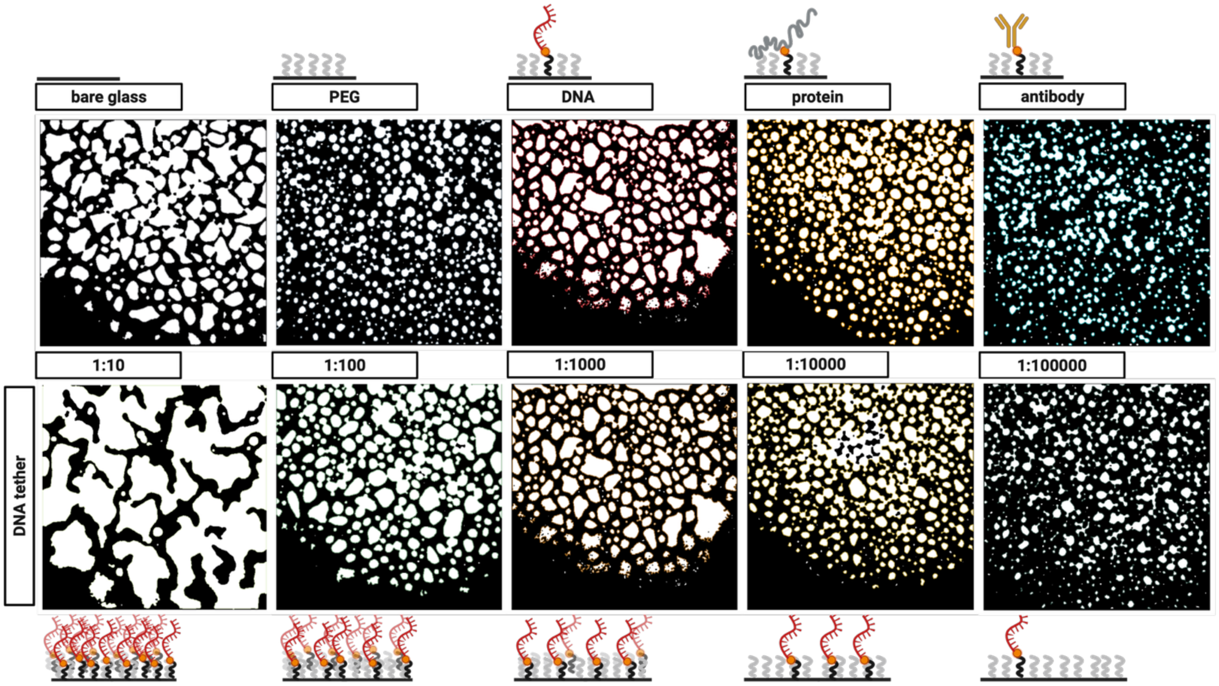

Figure S7 | Binary threshold images with drawn contours for circularity analysis. Full field of view is shown here and used for analysis in Fig. 1 b and d.
